# Anion-type modulates the effect of salt stress on saline lake bacteria

**DOI:** 10.1007/s00792-022-01260-5

**Published:** 2022-02-09

**Authors:** Bianka Csitári, Anna Bedics, Tamás Felföldi, Emil Boros, Hajnalka Nagy, István Máthé, Anna J. Székely

**Affiliations:** 1 Department of Microbiology, ELTE Eötvös Loránd University, Pázmány Péter stny. 1/c, 1117 Budapest, Hungary; 2 Department of Ecology and Genetics/Limnology, Uppsala University EBC, Norbyvägen 18D, 75236 Uppsala, Sweden; 3 Department of Microbiology, Tumor and Cell Biology, Karolinska Institutet, Solnavägen 9, 17165 Stockholm, Sweden; 4 Depatment of Molecular Ecology, Institute of Aquaculture and Environmental Safety, Hungarian University of Agriculture and Life Sciences, Páter Károly utca 1, 2100 Gödöllő, Hungary; 5 Institute of Aquatic Ecology, Centre for Ecological Research, Karolina u. 29, 1113 Budapest, Hungary; 6 Department of Bioengineering, Sapientia Hungarian University of Transylvania, Piaţa Libertăţii 1, 530104 Miercurea Ciuc, Romania; 7 Department of Aquatic Sciences and Assessment, Swedish University of Agricultural Sciences (SLU), Box 7050, 75007 Uppsala, Sweden

**Keywords:** Alkaline habitat, Bacterioplankton, Athalassic, Soda lake, Natronophiles, Salt stress

## Abstract

Beside sodium chloride, inland saline aquatic systems often contain other anions than chloride such as hydrogen carbonate and sulfate. Our understanding of the biological effects of salt composition diversity is limited; therefore, the aim of this study was to examine the effect of different anions on the growth of halophilic bacteria. Accordingly, the salt composition and concentration preference of 172 strains isolated from saline and soda lakes that differed in ionic composition was tested using media containing either carbonate, chloride or sulfate as anion in concentration values ranging from 0 to 0.40 mol/L. Differences in salt-type preference among bacterial strains were observed in relationship to the salt composition of the natural habitat they were isolated from indicating specific salt-type adaptation. Sodium carbonate represented the strongest selective force, while majority of strains was well-adapted to growth even at high concentrations of sodium sulfate. Salt preference was to some extent associated with taxonomy, although variations even within the same bacterial species were also identified. Our results suggest that the extent of the effect of dissolved salts in saline lakes is not limited to their concentration but the type of anion also substantially impacts the growth and survival of individual microorganisms.

**Supplementary Information:**

The online version contains supplementary material available at 10.1007/s00792-022-01260-5.

## Introduction

According to a conventional definition, saline lakes have a salinity of at least 3 g/L (Williams 1996). Inland saline lakes occur on every continent (Hammer 1986; Sorokin et al. 2014; Waiser and Robarts 2009), and their total number and volume on Earth is comparable to that of all freshwater lakes (Williams 1993; Lerman 2009). Athalassic saline lakes, that have not been connected to marine systems in geologically recent times, are globally widely distributed (De Wit 2016). They typically occur in dry (semiarid and arid) regions and have endorheic origin which means that they are located in hydrologically closed basins (Waiser and Robarts 2009; Boros and Kolpakova 2018). Endorheic lakes are filled with precipitation water, groundwater and runoff from the surrounding land but due to the lack of outflow, water loss happens only through evaporation, which leaves behind the dissolved salts (Wetzel 2001; Tundisi and Tundisi 2012). Besides the characteristics of the drainage area, several further processes including anthropogenic and climate drivers can contribute to the high and often variable salinity levels of lakes. For example, shallow saline lakes are particularly exposed to the effect of precipitation and evaporation induced water level fluctuation with droughts leading to drastic increases in salinity and even periodical complete desiccation (Hammer 1986; Waiser and Robarts 2009; Schagerl 2016; Szabó et al. 2020), while unrestricted water withdrawals from the watershed (e.g., irrigation) can also cause increases in salinity (Liu et al. 2020).

In terms of salt concentration and ionic composition, saline lakes may differ significantly from each other (Waiser and Robarts 2009; Boros and Kolpakova 2018). Saline waters can be characterized by the amount of eight major ions dissolved in the water: Ca^2+^, Mg^2+^, Na^+^, K^+^, HCO_3_^−^, CO_3_^2−^, Cl^−^ and SO_4_^2−^. Similarly to the salt concentration, chemical composition of the salts is also primarily determined by the mineral composition of the drainage basin. On the other hand, flow dynamics between the water body and the sediment as well as biological activities (e.g., human activities, such as the application of fertilizers in the surrounding agricultural area) can further influence chemical composition. The pH of lake water is also affected by the type of rock present in the drainage area. In the case of soda lakes, high concentration of dissolved carbonates increases pH and serves as a buffer creating a permanently alkaline character for the saline lake (Wetzel 2001; Boros and Kolpakova 2018). Furthermore, due to the evaporation induced concentration increases, certain types of salts may precipitate, while others remain in solution further modifying the ionic composition. The first salt to precipitate is typically lime (CaCO_3_), followed by dolomite [CaMg(CO_3_)_2_], then gypsum (CaSO_4_ × H_2_O), and finally other salts (Waiser and Robarts 2009; Schagerl 2016). Therefore, most saline waters have a predominance of Na^+^, and there are only a few lakes dominated by Ca^2+^ and Mg^2+^ cations. However, there is a remarkable diversity in the case of anions: although Cl^−^ anion dominates in most saline lakes, soda lakes are dominated by HCO_3_^−^ and CO_3_^2−^, while SO_4_^2−^ is also present in many saline lakes at relatively high concentration values (Wetzel 2001; Boros and Kolpakova 2018). Other elements in ionic forms, such as nitrogen, phosphorus, iron, manganese, silicon, have biological importance (e.g., in the case of lakes, the most important limiting factor of phytoplankton growth is phosphorus), but their contribution to total salinity is negligible (Wetzel 2001).

The concentration of different salts also affects other physicochemical properties of the lake water, e.g., with increasing salt concentration, the oxygen solubility and the freezing point of the water decreases, while density increases; and dissolved salts can also modulate the amount of available nutrients (Williams and Sherwood 1994; Shadrin 2018). However, salt concentration and composition also directly affect aquatic organisms. Increased salinity may reduce the abundance and diversity of macrophytes and simplify food web structures emphasizing the importance of planktonic species (Hammer and Heseltine 1988; Golubkov et al. 2018; Felföldi 2020). Hence, bacterioplankton play an essential role in the biochemical processes in saline aquatic habitats (Pedrós-Alió et al. 2000; Waiser and Robarts 2009; Schagerl 2016; Felföldi 2020). Simultaneously, bacteria due to their unicellular nature are particularly sensitive to osmotic and ionic changes making salt concentration and ionic composition a particularly important selective force modifying abundance, composition and activity of aquatic microbial communities (Székely and Langenheder 2014; Banciu and Muntyan 2015; Gunde-Cimerman et al. 2018; Menéndez-Serra et al. 2021). Despite the obvious importance of diverse ions in general cellular processes (Rosenberg et al. 2013), salinity tolerance studies usually focus only on the effect of overall salt concentration, which is mainly regarded as NaCl, or salinity and its values are estimated based on in situ measurements of electric conductivity. Although salinity can be easily calculated indirectly from conductivity, it depends not only on temperature but on the concentration of the actual ions (Williams and Sherwood 1994). This can be circumvented with site-specific empirical formulas (e.g., Keresztes et al. 2012; Boros et al. 2014), but the biotic and abiotic effects of individual major ions dissolved in the water are rarely considered (Williams and Sherwood 1994; Fox-Powell and Cockell 2018).

**Fig. 1:**
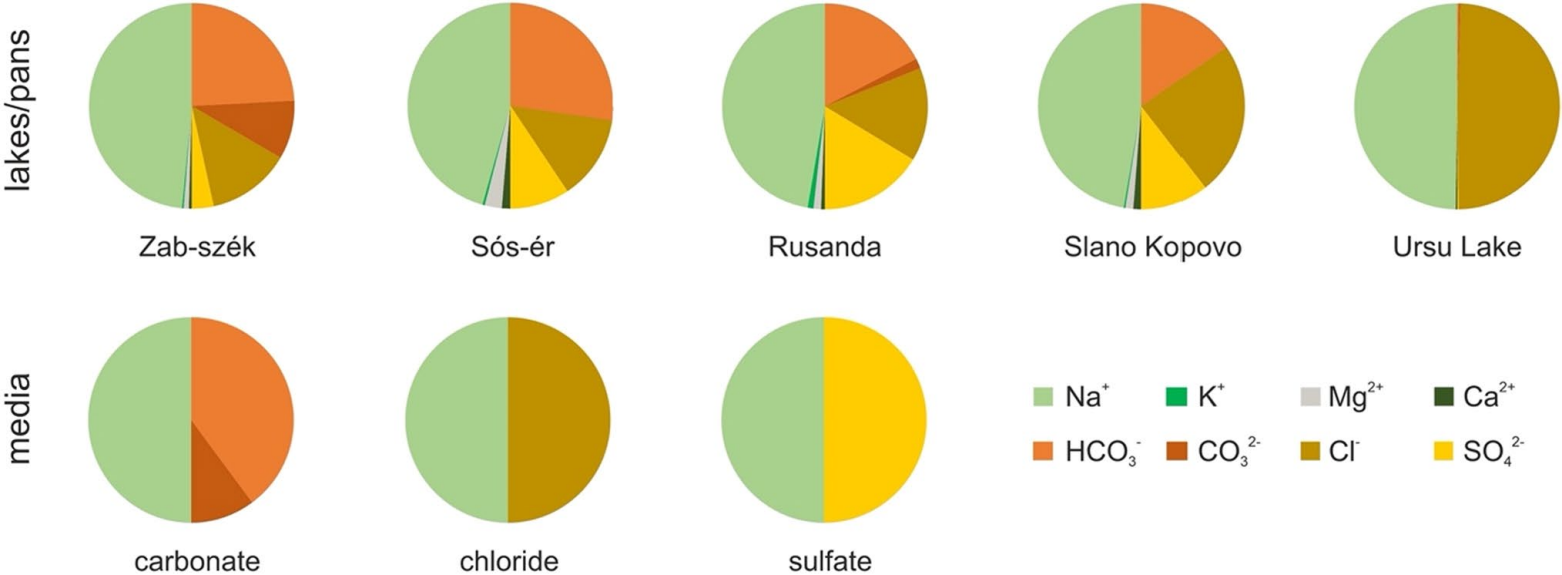
Salt composition of the lake water from where the strains were isolated and the media used in the salt tolerance test. Dissolved salts are shown in molar ratio. The ionic compositions of the lakes are based on data from Boros et al. (2014) and Andrei et al. (2015). Data for the ionic composition of Roşu Lake is not available, but as Roşu Lake is a tributary of Ursu Lake, the two lakes are expected to have fairly similar ionic composition

The aim of this study was to determine the effect of different concentrations of three anions (i.e., CO_3_^2−^ + HCO_3_^−^, Cl^−^ and SO_4_^2−^) common to saline lakes on bacterial strains isolated from such lakes. More specifically we hypothesized that the origin of the strains is related to their preferential adaptation to specific types of anions. To achieve this, growth tests were performed using media with different salt concentration values and ionic compositions on bacterial strains that were isolated from three sets of saline lakes that differed in ionic composition (Fig. 1): (1) two soda lakes located in Kiskunság (Hungary, Central Europe), characterized by high sodium hydrogen carbonate content (Boros et al. 2014); (2) two soda lakes in Vojvodina (Serbia, Central/Southeast Europe) with approximately equal proportion of sodium hydrogen carbonate, sodium chloride and sodium sulfate (Boros et al. 2014); and (3) two Transylvanian saline lakes (Romania, Eastern Europe), which are dominated by sodium chloride (Borsodi et al. 2013; Máthé et al. 2014; Andrei et al. 2015; Felföldi et al. 2016).

## Materials and methods

### Sample collection and determination of environmental variables

The bacterial strains used in this study were isolated from water samples collected from Zab-szék and Sós-ér soda pans located in Kiskunság (Hungary) on October 18, 2016, and from water samples collected from Rusanda soda pan and Slano Kopovo soda-saline pan (according to the classification Boros and Kolpakova 2018) in Vojvodina (Serbia) on May 17, 2018. In each case, pooled water samples were taken from 10 to 15 different points of the pans. Bacterial strains isolated from the upper layers of the saline Ursu Lake (Bear Lake) and Roşu Lake (Red Lake) in Transylvania (Romania) were obtained from the culture collection of the Department of Bioengineering, Sapientia Hungarian University of Transylvania (Miercurea Ciuc, Romania).

Environmental characteristics of the sampling sites in Kiskunság and Vojvodina were determined according to the methods described in detail by Pálffy et al. (2014), while in the case of the Transylvanian sites, literature data were used (Borsodi et al. 2013; Máthé et al. 2014; Andrei et al. 2015; Felföldi et al. 2016). The location of the sampling sites is shown in Figure S1 and the ionic composition of the water of the lakes in Fig. 1.

### Strain isolation and taxonomic identification

Water samples collected for strain isolation were processed within 24 h after collection. Lake water characteristics (salinity, ionic composition, pH, nutrient content) were considered in the design of the culture media used for strain isolation. Several different types of media were applied to isolate strains from the samples collected from Kiskunság (Table S1). Since the soda pans of the area are important refuges of aquatic and migratory birds, significant nutrient load from bird guano is expected to affect these sites (Boros et al. 2008, 2016). Therefore, some media contained the main components of guano such as uric acid and proteins (Nahm 2003), or contained specific organic compounds such as cellobiose and humic acid, as it has been shown that macrophytes and groundwater are the primary source of organic carbon in these pans (Boros et al. 2020). Szuróczki et al. (2020) suggested that the bacterioplankton of the soda lakes of this region preferentially utilize organic compounds, such as serine, asparagine, arginine and glycogen, thus two additional media were designed containing these compounds. All media applied for the cultivation from soda pan samples (Kiskunság and Vojvodina) contained CO_3_^2−^ and HCO_3_^−^ ions that also served as a buffer and provided a pH of 9.0–9.5. For bacterial strain isolation, the standard dilution plating technique was applied. The incubation was carried out at 15 or 20 °C in the case of Kiskunság and Vojvodina pans, respectively (temperatures similar to the values measured on site) for 7–21 days. Colonies with different morphologies were isolated and purified.

The genomic DNA from the strains was extracted by bead beating using sterile glass beads according to Vajna et al. (2016). The 16S rRNA gene was amplified by PCR using the primers 27F (5′ ‒ AGA GTT TGA TCM TGG CTC AG ‒ 3′) and 1492R (5′ ‒ GGT TAC CTT GTT ACG ACT T ‒ 3′) following the method described in detail previously (Kalwasińska et al. 2015). PCR products were purified and sequenced by LGC Genomics (Berlin, Germany). Chromatograms were manually corrected with Chromas (Technelysium Pty Ltd, South Brisbane, Australia) for the errors of automatic base calling. For taxonomic identification, EzBioCloud’s online service was used (Yoon et al. 2017). Strains were considered to be identified at species level above 97% 16S rRNA gene sequence similarity (Tindall et al. 2010). The GenBank accession numbers of the obtained sequences are: MK504162‒MK504333.

### Salt tolerance tests

Before the salt tolerance tests, strains were transferred and grown in liquid media that had the same composition as the solid media used for their maintenance (Table S1). After 7 days of incubation at room temperature (~ 25 °C), the absorbance of the liquid cultures was standardized to OD 0.2–0.3 with sterile broth using a Biolog 21,906 absorbance meter.

The salt tolerance tests were performed using nutrient-based (DSMZ medium 1, www.dsmz.de) media containing sodium chloride, sodium hydrogen carbonate and sodium carbonate or sodium sulfate at different concentration (Table 1); which throughout this study are referred to as ‘chloride’, ‘carbonate’ and ‘sulfate’ media, respectively. For each salt treatment, six different concentrations ranging from 0.06 to 0.40 mol/L were applied (Table 1). As the commonly used salinity categories according to Hammer (1986) are defined based on mass concentrations (w/v%), most of the applied salt concentrations corresponded to hypo- and mesosaline salinity. The only exception was the highest salt concentration treatment used for sulfate, which due to the high molar weight of sodium sulfate corresponded to the hypersaline category. To minimize the ‘natural’ buffering (i.e., pH increasing) effect of sodium carbonates, the pH of all test media was set to pH 9.0 ± 0.2 with 4 M NaOH solution. In summary, the tests were performed using nutrient broth containing one of the three tested salts and each type of media consisted of a series of eight different salt concentration values.

**Table 1:** Mass and molar concentration of salts in the liquid nutrient media used for the salt tolerance tests

Molar concentration	Mass concentration					
	Carbonate medium (NaHCO_3_ + Na_2_CO_3_**)		Chloride medium (NaCl)		Sulfate medium (Na_2_SO_4_)	
[mol/L]	[g/L]	Salinity category*	[g/L]	Salinity category*	[g/L]	Salinity category*
0.40	33.6	Mesosaline	23.4	Mesosaline	56.8	Hypersaline
0.28	23.2		16.1	Hyposaline	39.2	Mesosaline
0.19	16.0	Hyposaline	11.1		27.0	
0.13	11.0		7.7		18.6	Hyposaline
0.09	7.6		5.3		12.8	
0.06	5.2		3.6		8.8	

*According to the mass per volume (g/L) based classification of Hammer (1986)

**In a ratio of 8:1 (8 g/L NaHCO_3_ + 1 g/L Na_2_CO_3_), similarly to the ratio as they present naturally in the soda pans of the Carpathian Basin (Boros et al. 2014)

Salt tolerance tests were performed in 96-well microtiter plates using 300 μL of salt-containing nutrient medium and 10 μL of bacterial cell suspension. Sterile nutrient broth was used as a salt-free control. Two uninoculated wells served as negative controls on each microplate.

Microtiter plates were incubated at 20 °C, and the optical density (OD_590_) of broths was measured after 5, 12 and 21 days with a Tecan Sunrise microplate reader. In most of the cases, the absorbance values did not increase significantly after the fifth day of incubation (data not shown); therefore, data measured on that day were used for the subsequent analyses.

### Data analysis

To evaluate the differences in the anion preference of the strains, the OD values measured for each strain in the 19 different growth media were z-score normalized, and then the normalized OD values were multiplied by the molar concentration of each medium and summed within ion types to create a weighted growth value for each strain and anion type (i.e., carbonate, chloride and sulfate). The primary anion preference of each strain was determined based on the anion type of the media, where it showed the highest weighted growth. The impact of the region of origin on the weighted growth of each strain in the different anionic-type media was tested by Kruskal–Wallis test, while the different groups were compared with Dunn-test and *p* values were adjusted with the Benjamini–Hochberg method. A heatmap was generated to visualize the z-score normalized OD values and to explore the relationship of the k-mer clustering (k = 3) to the origin and taxonomical affiliation of the strains. The z-score normalization was performed using Microsoft Excel, while all statistical analyses and visualization were carried out in R (R Core Team 2017). The heatmap was generated using the ComplexHeatmap package.

## Results

### Ionic composition of the lakes

The physical and chemical parameters of the water samples are summarized in Table S2, and the ionic compositions of the studied lakes are presented in Fig. 1. All lakes contain > 90 e% (molar equivalent according to the separated calculation of total cation or anion pools) Na^+^ as cation. As anion the Transylvanian lakes have almost exclusively chloride (> 94 e%; with ~ 4 e% sulfate in the deeper regions of Ursu Lake), while the soda pans of the Kiskunság contain ~ 26–27 e% of chloride and ~ 55–65 e% of carbonates (hydrogen carbonate + carbonate). Compared to Kiskunság, the soda pans of Vojvodina have lower concentration of carbonates (~ 30–38 e%) and similar or higher values of chloride (29 and 48 e% in Rusanda and Slano Kopovo, respectively). The sulfate content of the water is the highest in the soda pans of Vojvodina (33 e% in Rusanda and 21 e% in Slano Kopovo) and moderate values are characteristic for the soda pans in the Kiskunság (19 e% in Sós-ér and 7 e% in Zab-szék).

### Strain isolation and taxonomic identification

The different growth media and solidifying agents used for the bacterial strain isolation from Kiskunság soda pans (Table S1) did not result in remarkable differences in colony counts (Csitári et al. 2018) and similar strains were isolated on different media. Therefore, in the case of the soda pans from Vojvodina only one type of medium was used for the isolation of strains.

**Fig. 2:**
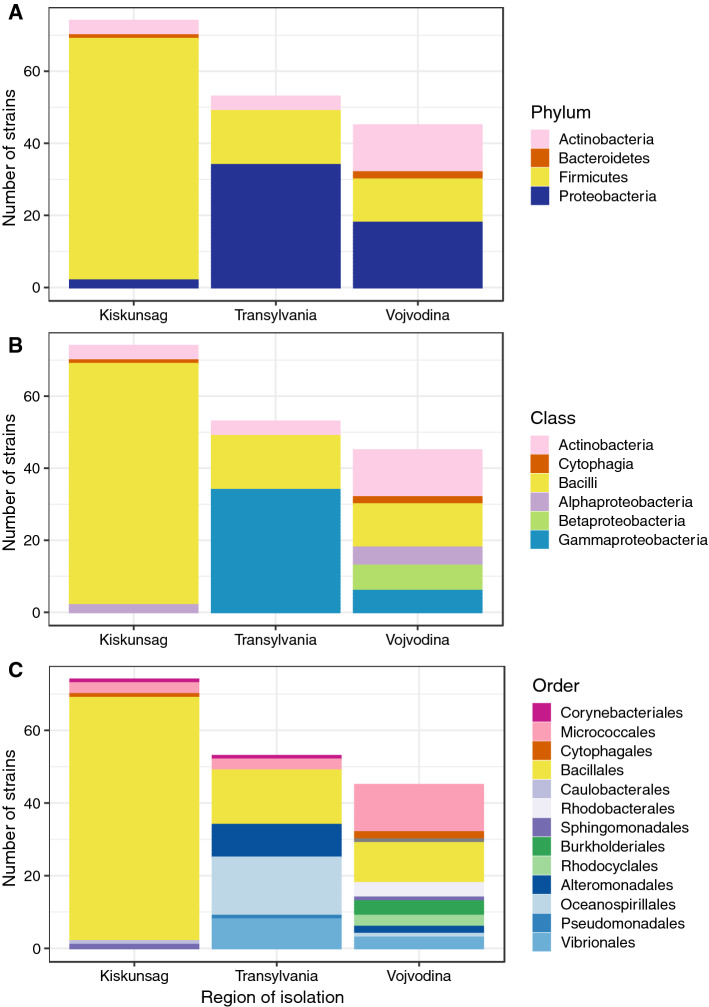
Taxonomic affiliation of the studied bacterial strains by region of isolation. Phylum- (**A**), class- (**B**) and order-level (**C**) distribution

A total of 172 bacterial strains were used in this study. Out of them, 119 were isolated within this study from Kiskunság and Vojvodina (74 and 45 strains, respectively), while 53 strains came from a culture collection containing strains that were previously isolated from Transylvania (Table S5). Most strains were identified at the species level as they showed > 97% pairwise nucleotide sequence similarity based on their 16S rRNA gene sequence with previously described species (Table S5). Some of the closest relatives of these species were actually first isolated and described from the same sites or other sites within the same regions, e.g., *Bacillus alkalisediminis* (Borsodi et al. 2011), *Nitrincola alkalilacustris* (Borsodi et al. 2017) and *Rhodococcus sovatensis* (Táncsics et al. 2017). Based on the results of taxonomic identification, the bacterial strains belonged to four phyla: Firmicutes, Proteobacteria, Actinobacteria and Bacteroidetes (Fig. 2A). These four phyla represented 6 classes, 13 orders and 34 genera (Fig. 2., Table S5). Almost all Firmicutes strains belonged to the Bacillales order with *Bacillus* and *Salipaludibacillus* being the most common genera (69 and 17 strains, respectively, out of 93). In general, almost half of the strains (48%) belonged to these genera and members of the order Bacillales had a remarkable contribution (> 24%) in each region. However, Bacillales were the most common strains only in the collections isolated from the Kiskunság soda pans (90% of Kiskunság strains), while among the strains from Vojvodina and Transylvania proteobacterial strains were present in greater numbers (64% and 40%, respectively). In the Transylvanian culture collection all proteobacterial strains belonged to Gammaproteobacteria, while in Vojvodina, Alpha-, Beta- and Gammaproteobacteria were represented almost equally (Fig. 2B). The Kiskunság collection contained only two Alphaproteobacteria strains and five further strains that did not belong to Bacilli class but to Actinobacteria and Cytophagia. The most diverse group of bacterial strains was isolated from the soda pans of Vojvodina with 18 different genera. In this collection, besides the proteobacterial and Bacillales strains, a substantial amount of actinobacterial strains (29%) were also present that were primarily represented by members of the *Nesterenkonia* genus.

### Salt tolerance tests

**Fig. 3:**
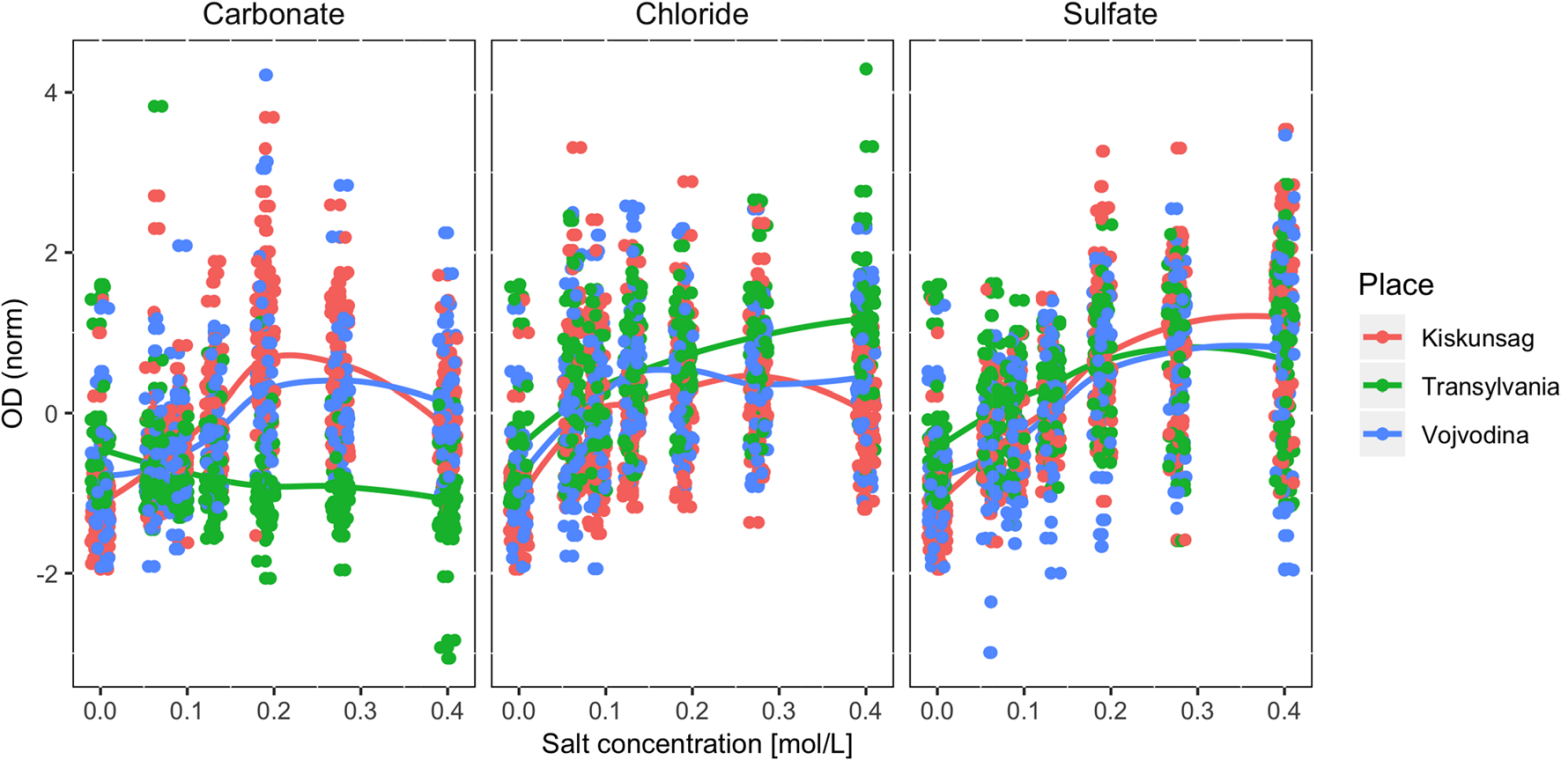
Growth intensity of bacterial strains in liquid media containing the three tested salt types in different concentration values based on turbidity data (z-score normalized OD). Trendlines are drawn according to mean OD values

In this study a total of 3268 individual salt tolerance measurements have been obtained using 172 bacterial strains grown in 19 different ionic composition and/or salt concentration media (6 carbonate, 6 chloride, 6 sulfate and 1 salt-free nutrient media as control). Based on the measured OD values, it can be generally concluded that the members of the studied bacterial collection grew more intensively at salt concentration values higher than the control medium (only 2 strains had their growth maximum in the control medium) (Fig. 3).

**Fig. 4:**
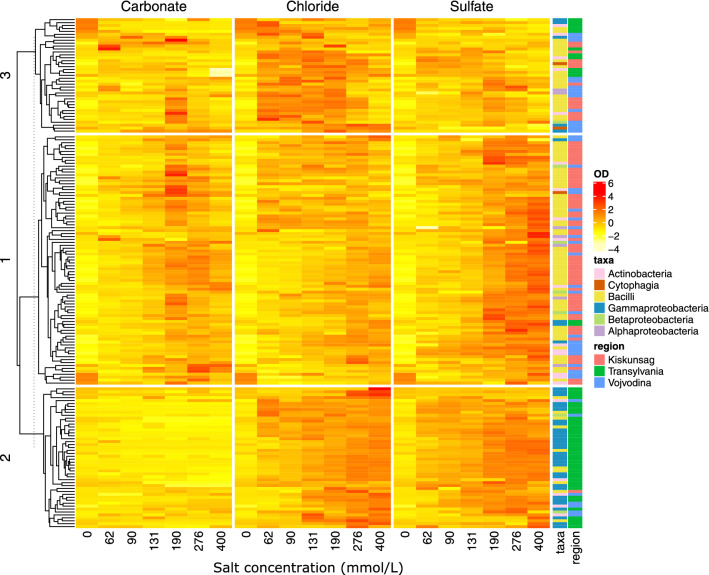
Clustering of the growth patterns measured in the salt tolerance test. Heatmap displays the z-score normalized OD values by anion type of the media. Dendrogram on the left shows hierarchical clustering by Euclidean distance clustered, colored bars on the right indicate taxonomic affiliation and the region of isolation of each studied bacterial strain

The three clusters of the heatmap showed distinctive growth patterns in the three different anion-type media (Fig. 4). Members of cluster 1 grew well in higher concentrations of the sulfate media, showed slight preference of intermediate concentrations of carbonate and grew relatively poorly in chloride media. In respect of the region of origin 52 of the 76 strains in this cluster were isolated from Kiskunság (28 from Sós-ér and 26 from Zab-szék), 22 from Vojvodina (15 from Rusanda and 7 from Slano Kopovo), and only three from Transylvania (2 from Ursu and 1 from Roşu Lake). Taxonomically all six classes were present in cluster 1 but Bacilli was the most abundant (47 strains) and the majority of Actinobacteria strains (11) also clustered into this group. In contrast, the strains in cluster 2 showed poor growth in carbonate media and intensive growth at higher concentrations of chloride and sulfate media. The majority of strains in this cluster were isolated from Transylvania (47 of 55 with 32 from Ursu and 15 from Roșu Lake), and only 7 and 1 from Vojvodina and Kiskunság, respectively. The majority of cluster 2 strains belonged to Gammaproteobacteria (32) followed by Bacilli (15), Actinobacteria (6), and Betaproteobacteria (2). Finally, cluster 3 comprised strains that showed the best growth at medium concentrations of chloride and relatively worse growth in carbonate and sulfate media. Most of the strains in this cluster were isolated from Kiskunság (21 with 9 from Sós-ér and 12 from Zab-szék) and Vojvodina (16 with 8 from Rusanda and 8 from Slano Kopovo) and only three from Transylvania. Taxonomically, cluster 3 was similar to cluster 1 with members of all six classes but the majority belonged to Bacilli (26) followed by Actinobacteria (4).

The salt preference determined based on the comparison of the weighted growth values of each strain in the three different salts showed that 59% of the strains grew best in sulfate, 33% in chloride and 8% in carbonate media (Table 2). The comparison of the anion preference of all strains and the preference of the strains from each region indicated differences between regions. Sulfate preferring strains were over-represented among the strains from Kiskunság (i.e., showed a higher ratio than expected based on the salt preference distribution of all strains: 72% of the strains from Kiskunság grew best on sulfate media, while only 59% of all strains), while they were slightly underrepresented among strains from Transylvania and Vojvodina. Chloride preference was over-represented among the strains from Transylvania (49% compared to 33% for all strains) and underrepresented for Kiskunság strains (20%). Finally, carbonate preference was over-represented among the strains from Vojvodina (18% compared to 8% for all) and missing among the Transylvanian strains (Table 2). Meanwhile, when it comes to the taxonomic affiliation of the strains, no substantial over- or underrepresentation was found for any of the most abundant taxa (i.e., taxa containing more than 10% of all strains) (Table S3).

**Table 2:** Primary anion preference of strains from different regions based on their maximum weighted growth value

Place of origin	All	Kiskunság	Transylvania	Vojvodina
All strains	172 (100%)	74 (100%)	53 (100%)	45 (100%)
Carbonate max	14 (8%)	6 (8%)	0 (0%)	8 (18%)
Chloride max	57 (33%)	15 (20%)	26 (49%)	16 (36%)
Sulfate max	101 (59%)	53 (72%)	27 (51%)	21 (47%)

**Fig. 5:**
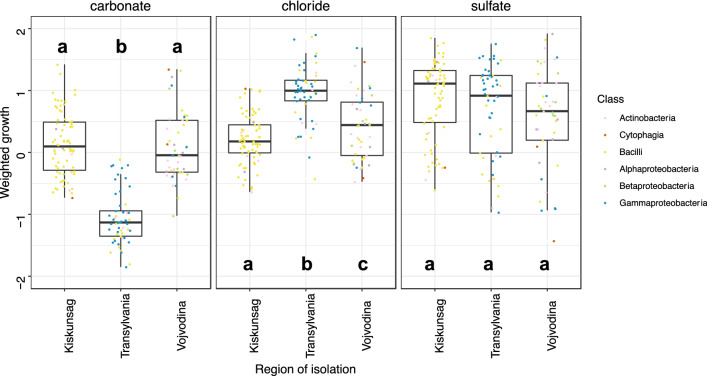
Weighted growth values of tested bacterial strains in different anion-type media by region of isolation. Data points colored by taxonomic affiliation of the corresponding strain. Boxplots show the first and the third quartiles with the median. Significantly different categories according to Dunn’s post-hoc test are marked by different letters

The mean weighted growth value of the strains also significantly differed among the three salts (Kruskal–Wallis chi-squared = 135.64, df = 2, *p* value < 0.001) with the growth in carbonate being substantially lower than in chloride and sulfate and the growth in sulfate being slightly but significantly higher than in chloride (Fig. 5, Table S4). The growth in the carbonate and chloride media significantly differed also for the strains originating from different regions (Kruskal–Wallis chi-squared = 90.823, df = 2, *p* value < 0.001, and Kruskal–Wallis chi-squared = 57.529, df = 2, *p* value < 0.001, respectively). Meanwhile, the weighted growth in the sulfate media did not show significant differences depending on the region of origin (Kruskal–Wallis chi-squared = 5.0544, df = 2, *p* value = 0.0799) (Fig. 5). The post-hoc analyses by Dunn-test revealed that overall the strains from Kiskunság and Vojvodina grew better in the carbonate media than the strains from Transylvania (Fig. 5, Table S4), while the strains from Transylvania grew better in chloride than those from Vojvodina and Kiskunság, and those from Vojvodina grew also slightly better in chloride than those from Kiskunság (Fig. 5). Furthermore, the taxonomic affiliation of the strains showed no substantial relationship with their weighted growth value in different anion media as no visible clustering by taxonomy along weighted growth in any salt could be identified (i.e., taxa belonging to the same class could show both high and low weighted growth for the same salt as seen in Fig. 5). In general, the bacterial strains of Kiskunság and Vojvodina are well-adapted to carbonate, chloride and sulfate, while bacteria isolated from Transylvanian lakes were the least adapted to carbonate, but grew well in the presence of chloride and sulfate.

## Discussion

In this study we demonstrated that the salt tolerance of strains isolated from saline lakes with different salt composition depends not only on the concentration of salt in the media but also the anion composition of the salt. Furthermore, the growth pattern and anion preference of strains was related to the anion composition of their source habitat. Namely, the majority of bacterial strains isolated from lakes dominated by chloride, were adapted to grow in media with higher concentrations of chloride, while strains isolated from aquatic systems containing a considerable amount of carbonate salts were adapted to grow with carbonates. On the other hand, the growth of most strains was supported by sulfate irrespective of their origin.

Contrary to most halotolerance studies focusing on the limits of growth, our study investigated growth at less extreme salt concentration values (0.06–0.4 M) such as those characteristic to the soda pans and the upper layers of the saline Transylvanian lakes from, where the studied bacterial strains were isolated. The tested salinity levels correspond mostly to hypo- and mesosaline conditions with the only exception of the highest sodium sulfate media that corresponded to hypersaline conditions. This discrepancy in the salinity category of the different anion media resulted from the alignment of the different media based on the molar concentration of the anions rather than the salinity unit expressed in mass (i.e., g/L). The reason for this choice was that both the osmotic stress and the most common haloadaptive strategies in microbes (e.g., expulsion of ions through the plasma membrane via ion channels and transporters; Banciu and Muntyan 2015) are defined by molar rather than mass concentrations. More precisely, in moderately saline aquatic habitats and in environments with fluctuating salinity such as those typical for shallow soda pans, microbes are more likely to cope with osmotic stress by applying the energetically more expensive but flexible ‘salt-out’ (’compatible solute’) strategy (i.e., exclusion of salt ions from the cytoplasm with simultaneous accumulation of low molecular weight organic compounds to lower the intracellular water activity and stabilize the hydration shell of proteins) rather than the ‘salt-in’ strategy (accumulation of inorganic salts intracellularly) (Oren 2013a; Gunde-Cimerman et al. 2018; Menéndez-Serra et al. 2021). Our study did not intend to elucidate which of the above strategies are applied by the studied strains; however, we know that the studied strains were either anoxygenic phototrophs (*Rhodobaca* and *Roseinatronobacter*) or heterotrophs (all other strains). Glycine betaine, a common compatible solute in prokaryotes, can be synthetized de novo by some phototrophs, while most halophilic heterotrophic bacteria possess systems that enable direct uptake of either betaine or its precursors such the amino acid glycine or choline (Gunde-Cimerman et al. 2018; Song et al. 2020). Pathways of glycine betaine synthesis from precursors have been described in various strains that belong to genera that were also present in our strain collection (e.g., *Bacillus*, *Halobacillus*, *Staphylococcus*; Rosenstein et al. 1999; Burkhardt et al. 2009; Nau-Wagner et al. 2012). According to the salt tolerance test almost all strains in this study preferred elevated salt concentrations over the control medium and all media used in this study contained complex components (e.g., peptone and meat extract) that most likely included the precursor molecule(s) of glycine betaine biosynthesis (Imhoff and Rodriguez-Valera 1984; Gunde-Cimerman et al. 2018) suggesting that the compatible solute strategy could be a common coping mechanism among our halophilic strains.

Natural and artificial saline aquatic habitats represent an enormous variability of salinity and ionic composition (brackish river estuaries, soda lakes, saltern crystallizer ponds, etc.; Hammer 1986; Waiser and Robarts 2009; Oren 2013a). Examples of saline systems with non-NaCl dominated ionic composition include soda lakes and ponds (with large amounts of Na-carbonates; Kebede et al. 1994; Boros et al. 2014; Schagerl 2016; Boros and Kolpakova 2018), MgSO_4_- and CaCl_2_-brines (Dickson et al. 2013; Fox-Powell and Cockell 2018) and soda lime deposits (mainly with calcium as cation; Kalwasińska et al. 2015, 2019). The Dead Sea also contains higher molar concentration of magnesium than sodium ions (Oren 2013b). Apart from saline surface waters, aquatic habitats with diverse salinity composition exist also beneath the surface of Earth such as in the deep anoxic brines of the Mediterranean Sea that are almost saturated with MgCl_2_ (Cita 2006) or the Ca- and K-dominated subsurface saline waters in sedimentary basins (Hanor 1994). Furthermore, non-NaCl dominated saline brines exist also on other planetary bodies, e.g., on Europa and Mars (Kargel 2000; Vaniman et al. 2004). Despite this huge diversity of ionic composition in saline aquatic habitats, most salt adaptation studies focus only on the biological effect of different concentration values of NaCl, making studies like ours, where salt tolerance is explored for salts others than NaCl crucial for the understanding of the selective forces that saline habitats exert on aquatic microbes.

Although organisms living in saline environments with different dominant ions are expected to have different strategies to cope with the unfavorable effects of dissolved ions (Hallsworth et al. 2007; Banciu and Muntyan 2015; Sorokin et al. 2015), the influence of different salts on bacteria has been scarcely studied previously. For example, Stevens and Cockell (2020) determined for a single *Bacillus subtilis* strain in eight different salts the highest molar concentration, where growth could be still detected. This limit concentration was 2.5 times higher for NaCl than for Na_2_SO_4_. Similarly, Fox-Powell and Cockell (2018) observed the shortest doubling times of a *Marinococcus* strain in sodium and chloride ion containing media. Both studies concluded that physicochemical properties such as ionic strength and water activity alone did not determine the limits of growth, instead the combination of anions and cations also had an effect on bacteria. Waajen et al. (2020) also indicated that anion properties were among the most important parameters that affected the survival of a *Planococcus halocryophilus* strain at extremely low temperature values when comparing brine solutions of nine different salts, while Banciu et al. (2004) observed higher biomass yield of a *Thialkalivibrio halophilus* strain in NaCl than in Na_2_CO_3_, and hypothesized that the adaptation to high salinities could, at least partly, be explained with differences in the osmotic pressure caused by these salts at equal sodium ion concentration (4 M). Contrary to these experiments performed on individual bacterial strains, to the best of our knowledge, systematic study on the effect of different dissolved ions on a large set of bacteria isolated from different saline lakes like ours (i.e., 172 bacterial strains isolated from 6 different lakes in 3 different regions and tested on 3 different salts) has not been carried out before.

Since most natural saline lakes have sodium cation dominance (Hammer 1986; Waiser and Robarts 2009), we focused only on the effect of dissolved anions (namely, chloride, sulfate and carbonates). We have to emphasize that as high concentration of dissolved Na-carbonates increases pH, to avoid the potential confounding effect of varying pH, all salt tolerance tests were performed under alkaline conditions (pH ~ 9.0), Still, carbonate had the most substantial effect on the growth, as on average the strains showed significantly poorer growth in these media than in chloride or sulfate indicating widespread maladaptation to carbonate. The special effect of carbonate on microbial life has been recognized before and Banciu and Sorokin (2013) even suggested distinguishing microorganisms that prefer Na-carbonates as natronophiles over NaCl-preferring halophiles. According to this nomenclature, only 14 strains in this study were truly natronophiles having higher growth in carbonate than in chloride or sulfate media. Still, interestingly none of these strains had its highest growth in the highest carbonate concentration media but at intermediate concentrations suggesting that coping with high carbonate concentrations is challenging even for carbonate adapted microorganisms.

The anion-preference and tolerance of the studied strains was related to whether they were isolated from chloride- (Transylvania region: Ursu and Roşu Lake) or carbonate-rich saline lakes (Kiskunság: Zab-szék and Sós-ér; Vojvodina: Rusanda and Slano Kopovo). First, all natronophiles were isolated from the carbonate-rich lakes. Next, most strikingly the strains isolated from the NaCl-dominated saline lakes that had only negligible proportion (~ 1 e%) of carbonates, grew very poorly on carbonate further corroborating the idea that coping with carbonates requires special physiological adaptation. The strains isolated from the Transylvanian lakes had significantly better growth in chloride than the strains isolated from the carbonate-rich lakes and the NaCl-preferring halophilic strains were also over-represented among them. Meanwhile, on average the strains grew best in the sulfate media and except for a slight over-representation of sulfate preference among the strains isolated from Kiskunság, no regional differences could be detected for this salt.

All source habitats contained a remarkable amount of sulfate (4–33 e%), which could originate both from decomposing organic matter (e.g., from leaves of surrounding trees or shoreline macrophytes), as well as from dissolution from the bedrock (in the case of Ursu Lake, sulfate concentration increases gradually with depth probably due to the activity of green sulfur bacteria; Máthé et al. 2014; Andrei et al. 2015; Felföldi et al. 2016; Alexe et al. 2017). Sulfate can both act as an essential nutrient and at high concentration values as an osmotic stressor of organisms (Mera et al. 2016). After phosphate, sulfate is the second most abundant soluble oxyanion inside the bacterial cells (Silver and Walderhaug 1992). Furthermore, sulfur accounts for 0.9–1.4% of cellular dry matter content, and it has a key role in cells as a component of various amino acids and coenzymes (Overmann 2013). Previous studies with single bacterial strains showed higher tolerance or preference of NaCl over Na_2_SO_4_ (Quesada et al. 1987; Fox-Powell and Cockell 2018; Stevens and Cockell 2020). Contrary to this, in our study, the majority of strains irrespective of their site of isolation grew best in the sulfate-containing media and not in chloride. The intensive growth of most tested strains in the presence of sulfate suggest proper adaptation to high concentrations of this anion. This could be explained with the abundant occurrence of sulfate in nature as it is the most thermodynamically stable form of sulfur under oxic conditions (Rabus et al. 2013) as well as with the importance of sulfate to microorganisms as the most common source of sulfur (Aguilar-Barajas et al. 2011).

Overall bacterial strain collections isolated from all sites showed a broad range of salt adaptation. For example, strains from the two soda lake regions showed growth at diverse concentrations of all three tested salts, while the strains from Transylvania, although they grew mostly poorly in carbonate media, had a wide optimum range for chloride and sulfate. This reflects the spatial and temporal variability in the salinity of the origin sites of the strains. The salt concentration of the soda pans in the Carpathian Basin usually varies between 1 and 20 g/L with substantial seasonal variability and occasional extremes as high as 70 g/L (Boros et al. 2014, 2017). In the case of Ursu Lake, characteristic vertical gradients and changes in the salinity and chemical composition has been observed, and human recreational activities (mixing related to bathing) during the summer period can also slightly alter the concentration and distribution of ions in the different layers of the lake (Máthé et al. 2014; Andrei et al. 2015; Felföldi et al. 2016). The broad and diverse salt tolerance among the strains of each habitat suggests that the local microbial communities are well-adapted and resilient to the inherent environmental variation of the source lakes.

It should be mentioned that there was an association between the observed differences in salt tolerance and the taxonomic affiliation of the strains. Accordingly, we found that adaptation to carbonate was more common for Bacilli strains than for other taxa, while carbonate maladaptation was more often detected among proteobacterial and especially Gammaproteobacteria strains. Obviously, salinity related adaptive traits have a phylogenetic component that is reflected in taxonomy (Morrissey and Franklin 2015). Accordingly, there was also a marked difference in the taxonomic composition of the strain collections isolated from the three different regions indicating the differences in selective forces present at the source habitats. This is not surprising as salinity is well-known for being one of the most important environmental factors defining the composition of microbial communities through phylogenetically clustered salinity preference (Tamames et al. 2010; Herlemann et al. 2016; Székely and Langenheder 2014). Although, when evaluating the taxonomic composition of the strain collections it should be considered that only a small proportion of bacteria from a given community can be cultivated (Puspita et al. 2012) and much greater diversity can be detected with culture-independent DNA-based methods (Newton et al. 2011). Therefore, the composition of the strain collection should be taken only as a glance of a very limited set of the true microbial diversity of the sampling sites. For more realistic overview, culture-independent analyses of the microbial communities of the source lakes have been previously published (Borsodi et al. 2013; Máthé et al. 2014; Andrei et al. 2015; Felföldi et al. 2016; Szabó et al. 2017, 2020).

Meanwhile, interspecies variability in response to salt stress was also observed with strains of the same species exhibiting different traits. For example, the 9 strains of *Bacillus alkalisediminis* isolated from Zab-szék in Kiskunság clustered into the three different clusters on the heatmap and among them all three salt preferences types could be identified (i.e., carbonate, chloride and sulfate). The existence of high diversity of salt preference and tolerance within each class was also evident based on the weighted growth comparisons, while similar traits were identified in taxonomically distant taxa as indicated by the occurrence of natronophily in all six classes present among the strains of this study. In summary, although our results underpin the idea that salinity is a strong selective force and further emphasize the importance of anion type, we also showed that salinity stress traits are not deeply phylogenetically conserved and may vary even within the same bacterial species or genus.

## Conclusions

Our study demonstrated that the analysis of the effect of low to medium salinity stress exerted by different sodium salts (carbonate, chloride, and sulfate) can reveal rich, origin-dependent diversity in bacterial adaptation that studies testing salt tolerance and adaptation only to sodium chloride at high concentrations mostly overlook. The results of our study, similar to the conclusions of previous studies based on the analysis of single strains, corroborated the idea that salinity tolerance is not only salt concentration dependent but it is also greatly influenced by the anion type of the given salt. More precisely, we concluded that in general sodium carbonate salts pose a selective force that impact bacteria more substantially than the other tested salts and to which only few, so called natronophilic microorganisms are adapted. Furthermore, we found that while sulfate tolerance was widespread among strains isolated from different saline sources, in general, growth intensity in media with different concentrations of different salts was related to the origin and to some extent the taxonomy of the strains. Understanding the impact of salinity stress caused by different anions on microbial growth and survival is particularly important considering the vast ionic diversity of athalassic saline lakes as well as the expected changes in salinity in such habitats due to climate change intensified evaporation and anthropogenic water withdrawal practices.

## Supplementary Information

Below is the link to the electronic supplementary material.

Supplementary file1 (DOCX 134 KB)
