# Supplementary material for: Anion-type modulates the effect of salt stress on saline lake bacteria
Source: Extremophiles. 2022 Feb 9;26(1):12. doi: 10.1007/s00792-022-01260-5 (PMC8825391; doi:10.1007/s00792-022-01260-5)
Supplement: Supplementary file 1 — Supplementary file1 (DOCX 134 KB) [file 792_2022_1260_MOESM1_ESM.docx]

SUPPLEMENTARY MATERIAL

**Anion type modulates the salt tolerance of bacterial growth in saline lakes**

Bianka Csitári, Anna Bedics, Tamás Felföldi, Emil Boros, Hajnalka Nagy, István Máthé, Anna J. Székely

**Supplementary Figure 1.** Geographic location of the isolation sources of bacterial strains. (A) Kiskunság (Sós-ér and Zab-szék); (B) Vojvodina (Rusanda and Slano Kopovo); (C) Transylvania (Ursu Lake and Roşu Lake).


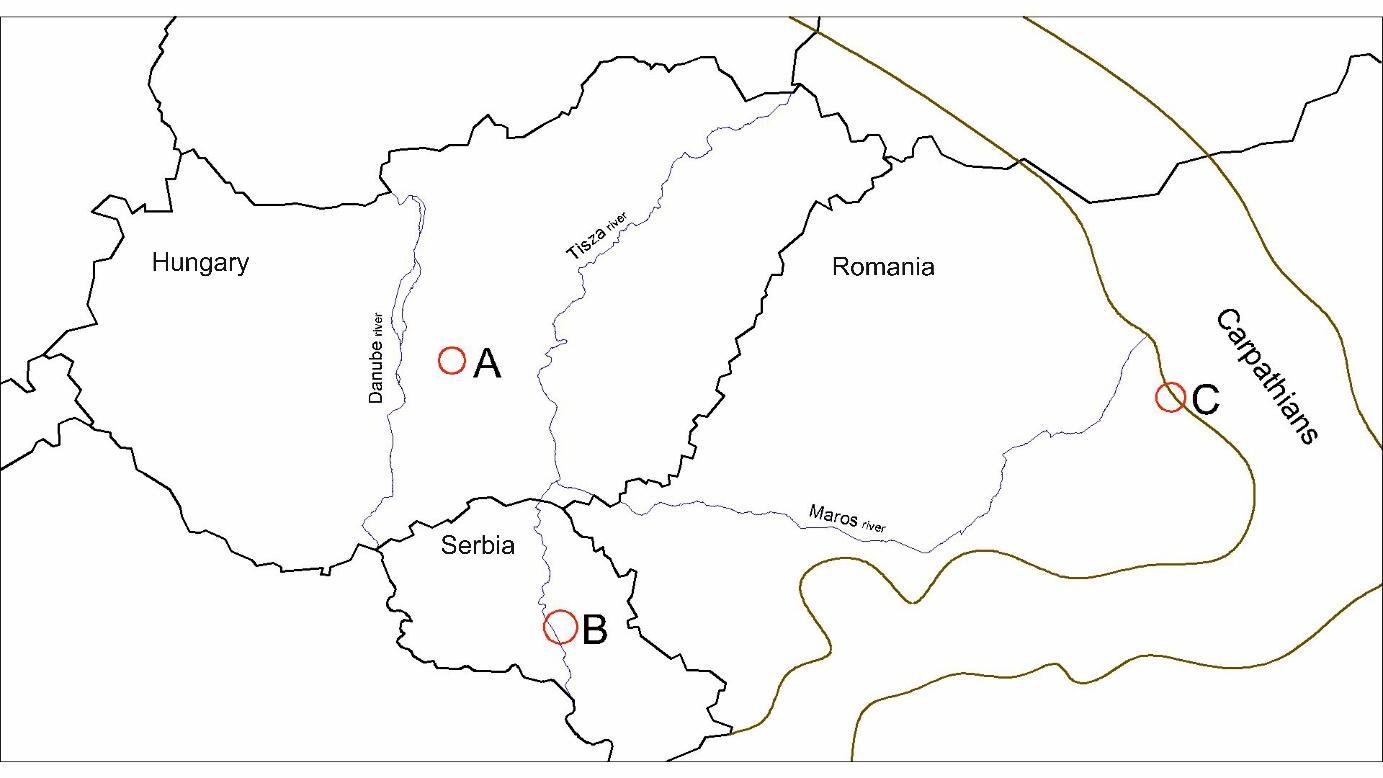


**Supplementary Table 1.** Composition of media used for strain isolation and maintenance. The last character of medium code in the case of the Kiskunság soda pan strains refers to the solidifying agent (A: 20 g/L agar or G: 14 g/L gellan gum with 0.75 g/L MgSO_4_ × 7 H_2_O and 0.5 g/L CaCl_2_ × 2 H_2_O for proper solidification), while the first character(s) refers to medium type (P: proteinaceous, E: excrement-like, A: amino acid-containing, H: humic acid-containing, MR2: modified R2A medium). The composition of soda basal medium (*SBM*): 8 g/L NaHCO_3_, 2.7 g/L Na_2_CO_3_ × 10 H_2_O, 1 g/L NaCl and 0.5 g/L KH_2_PO_4_ (final concentration). Components were dissolved in 1 L of water. n.a. – not applicable (strains were obtained from the culture collection of Department of Bioengineering, Sapientia Hungarian University of Transylvania, Romania).

| **Isolation of media** | | | | | | **Maintenance media** |
| --- | --- | --- | --- | --- | --- | --- |
| **Kiskunság** | **PA, PG** | **EA, EG** | **AA, AG** | **HA, HG** | **MR2A, MR2G** |  |
|  | 0.5 g peptone  1 g glucose  0.5 g yeast extract  0.2 g NaNO_3_  0.2 g NH_4_Cl  0.5 g KH_2_PO_4_  agar or gellan gum  *SBM*  **pH 9.5** | 0.5 g peptone  0.5 g uric acid  0.2 g NH_4_Cl  0.5 g KH_2_PO_4_  agar or gellan gum  *SBM*  **pH 9.5** | 0.2 g L-serine  0.25 g L-asparagine  0.35 g L-arginine  0.75 g glycogen  0.5 g KH_2_PO_4_  agar or gellan gum  *SBM*  **pH 9.5** | 1 g D-cellobiose  0.5 g humin acid  0.5 g KH_2_PO_4_  agar or gellan gum  *SBM*  **pH 9.5** | 0.5 g yeast extract  0.5 g proteose peptone  0.5 g kasamino acid  0.5 g glucose  0.5 g soluble starch  0.3 g sodium pyruvate  0.3 g KH_2_PO_4_  0.05 g MgSO_4_  agar or gellan gum  *SBM*  **pH 9.5** | 5 g peptone  3 g beef extract  8 g NaHCO_3_  2.7 g Na_2_CO_3_ × 10 H_2_O  1 g NaCl  0.5 g KH_2_PO_4_  15 g agar  **pH 9.5** |
| **Vojvodina** | 5 g peptone  3 g beef extract  5 g Na_2_SO_4_  2.5 g NaCl  2.5 g NaHCO_3_  15 g agar  **pH 9.0** | | | | | |
| **Transylvania** | n.a. | | | | | 5 g peptone  3 g beef extract  50 g NaCl  15 g agar  **pH 9.0** |

**Supplementary Table 2.** Location and environmental parameters of the isolation source lakes at the time of sampling. n.a. – not applicable (strains were obtained from the culture collection of Department of Bioengineering, Sapientia Hungarian University of Transylvania, Romania)

| **Lake**  **(Region)** | **Geographical coordinates** | **Date of sampling** | **Water depth (cm)** | **Secchi-disc transparency (cm)** | **Temperature (°C)** | **pH** | **Salinity (g/L)** |
| --- | --- | --- | --- | --- | --- | --- | --- |
| Zab-szék  (Kiskunság) | 46.829055  19.175115 | 18 Oct 2016 | 15 | 2.5 | 10.8 | 9.6 | 5.0 |
| Sós-ér  (Kiskunság) | 46.789017  19.144650 | 18 Oct 2016 | 22 | 10.5 | 11.0 | 9.3 | 7.2 |
| Rusanda  (Vojvodina) | 45.526986  20.297049 | 17 May 2018 | 15 | 2.0 | 23.9 | 9.6 | 15.0 |
| Slano Kopovo  (Vojvodina) | 45.628891  20.204604 | 17 May 2018 | 20 | 2.0 | 19.0 | 9.0 | 3.9 |
| Ursu Lake  (Transylvania) | 46.603792  25.085541 | n.a. | 1820* | 196 | n.a. | 7.1* | 5-320* |
| Roşu Lake  (Transylvania) | 46.606408  25.084397 | n.a. | 80** | - | n.a. | 8.9** | 160** |

* Data from Máthé *et al.* (2014); Felföldi *et al.* (2016); there is a vertical salinity gradient in the lake

** Data from Borsodi *et al.* (2013)

**Supplementary Table 3.** Primary anion preference of the strains belonging to the most abundant taxonomic categories (i.e. taxa containing more than 10% of all strains) based on their maximum weighted growth value.

| **Taxa** | **All** | **Actinobacteria** | **Micrococcales** | **Bacilli** | **Bacillaceae** | **Bacillus** | **Proteobacteria** | **Gammaproteobacteria** |
| --- | --- | --- | --- | --- | --- | --- | --- | --- |
| **All strains** | 172 (100%) | 21 (100%) | 19 (100%) | 94 (100%) | 90 (100%) | 66 (100%) | 54 (100%) | 40 (100%) |
| **Carbonate max** | 14 (8%) | 1 (5%) | 1 (5%) | 8 (9%) | 7 (8%) | 5 (8%) | 4 (7%) | 2 (5%) |
| **Chloride max** | 57 (33%) | 8 (38%) | 7 (37%) | 28 (30%) | 26 (29%) | 24 (36%) | 19 (35%) | 14 (35%) |
| **Sulfate max** | 101 (59%) | 12 (57%) | 11 (58%) | 58 (62%) | 57 (63%) | 37 (56%) | 31 (57%) | 24 (60%) |

**Supplementary Table 4.** Results of post-hoc analyses by Dunn-test of the weighted growth values of the strains comparing the three different salts and the different regions by each salt. P-value adjustment by the Benjamini-Hochberg method. Significant adjusted p-values (p < 0.05) are in bold.

A: Comparison weighted growth of all strains in different anion type test media.

| **Comparison** | **Z** | **P.unadj** | **P.adj** |
| --- | --- | --- | --- |
| carbonate - chloride | 8.0994 | 5.5251e-16 | **8.2876e-16** |
| carbonate - sulfate | -11.297 | 1.3510e-29 | **4.0529e-29** |
| chloride - sulfate | -3.1981 | 1.3834e-03 | **1.3834e-03** |

B: Comparison of weighted growth of strains from different isolation regions in the three anion type test media.

|  | **Carbonate** | | | **Chloride** | | | **Sulfate** | | |
| --- | --- | --- | --- | --- | --- | --- | --- | --- | --- |
| **Comparison** | **Z** | **P.unadj** | **P.adj** | **Z** | **P.unadj** | **P.adj** | **Z** | **P.unadj** | **P.adj** |
| Kiskunsag - Transylvania | 8.9376 | 3.9780e-19 | **1.1934e-18** | -7.5117 | 5.8363e-14 | **1.7509e-13** | 1.3616 | 0.17332 | 0.2600 |
| Kiskunsag - Vojvodina | 0.5148 | 6.0670e-01 | 6.0670e-01 | -2.0201 | 4.3371e-02 | **4.3371e-02** | 2.1826 | 0.0291 | 0.0872 |
| Transylvania - Vojvodina | -7.4540 | 9.0521e-14 | **1.3578e-13** | 4.7844 | 1.7147e-06 | **2.5720e-06** | 0.8267 | 0.4084 | 0.4084 |

**Supplementary Table 5.** Taxonomic affiliation of the bacterial strains used in this study based on 16S rRNA gene sequence similarity

| **Stain code** | **Isolation source** | **Sequence length (nt)** | **GenBank**  **Acc. No.** | **Closest related type strain(s)** | **Similarity**  **(%)** | **Higher Taxonomic rank** |
| --- | --- | --- | --- | --- | --- | --- |
| Vf2-S025 | Roşu Lake (Transylvania) | 1024 | MK504162 | *Salinivibrio costicola* | 99.90 | Proteobacteria; Gammaproteobacteria; Vibrionales; Vibrionaceae |
| Vecho-009B | Roşu Lake (Transylvania) | 991 | MK504163 | *Bacillus vietnamensis* | 99.60 | Firmicutes; Bacilli; Bacillales; Bacillaceae |
| Visz4-H004 | Roşu Lake (Transylvania) | 1087 | MK504164 | *Bacillus hwajinpoensis* | 99.54 | Firmicutes; Bacilli; Bacillales; Bacillaceae |
| Vf-10 | Roşu Lake (Transylvania) | 1029 | MK504165 | *Halomonas andesensis/*  *axialensis* | 99.22 | Proteobacteria; Gammaproteobacteria; Oceanospirillales; Halomonadaceae |
| Vf-18 | Roşu Lake (Transylvania) | 988 | MK504166 | *Salinivibrio costicola* | 100 | Proteobacteria; Gammaproteobacteria; Vibrionales; Vibrionaceae |
| Vf-S043 | Roşu Lake (Transylvania) | 1017 | MK504167 | *Chromohalobacter beijerinckii* | 99.80 | Proteobacteria; Gammaproteobacteria; Oceanospirillales; Halomonadaceae |
| Vecho-005B | Roşu Lake (Transylvania) | 1002 | MK504168 | *Bacillus vietnamensis* | 99.60 | Firmicutes; Bacilli; Bacillales; Bacillaceae |
| Vf2-S012 | Roşu Lake (Transylvania) | 980 | MK504169 | *Halomonas janggokensis* | 99.49 | Proteobacteria; Gammaproteobacteria; Oceanospirillales; Halomonadaceae |
| Vf2-S013 | Roşu Lake (Transylvania) | 1056 | MK504170 | *Halomonas gomseomensis* | 99.43 | Proteobacteria; Gammaproteobacteria; Oceanospirillales; Halomonadaceae |
| Vf4-S041 | Roşu Lake (Transylvania) | 968 | MK504171 | *Halomonas janggokensis* | 100 | Proteobacteria; Gammaproteobacteria; Oceanospirillales; Halomonadaceae |
| Vecho-012A1 | Roşu Lake (Transylvania) | 1031 | MK504172 | *Bacillus hwajinpoensis* | 99.52 | Firmicutes; Bacilli; Bacillales; Bacillaceae |
| VF2-S024 | Roşu Lake (Transylvania) | 1049 | MK504173 | *Halomonas andesensis* | 99.43 | Proteobacteria; Gammaproteobacteria; Oceanospirillales; Halomonadaceae |
| Vecho-012A2 | Roşu Lake (Transylvania) | 993 | MK504174 | *Bacillus hwajinpoensis* | 99.50 | Firmicutes; Bacilli; Bacillales; Bacillaceae |
| VI1-S011 | Roşu Lake (Transylvania) | 1032 | MK504175 | *Bacillus safensis/*  *zhangzhouensis* | 100 | Firmicutes; Bacilli; Bacillales; Bacillaceae |
| Vecho-012B1 | Roşu Lake (Transylvania) | 1134 | MK504176 | *Bacillus hwajinpoensis* | 99.56 | Firmicutes; Bacilli; Bacillales; Bacillaceae |
| Vecho-005A | Roşu Lake (Transylvania) | 1035 | MK504177 | *Bacillus vietnamensis* | 99.61 | Firmicutes; Bacilli; Bacillales; Bacillaceae |
| Vecho-012B2 | Roşu Lake (Transylvania) | 1087 | MK504178 | *Bacillus hwajinpoensis* | 99.54 | Firmicutes; Bacilli; Bacillales; Bacillaceae |
| Vecho-003B | Roşu Lake (Transylvania) | 1070 | MK504179 | *Pseudomonas sabulinigri* | 99.16 | Proteobacteria; Gammaproteobacteria; Pseudomonadales; Pseudomonadaceae |
| MIII2-H001 | Ursu Lake (Transylvania) | 1063 | MK504180 | *Marinobacter adhaerens* | 99.91 | Proteobacteria; Gammaproteobacteria; Alteromonadales; Marinobacter_f |
| MVIII4-B | Ursu Lake (Transylvania) | 1048 | MK504181 | *Halomonas arcis* | 99.04 | Proteobacteria; Gammaproteobacteria; Oceanospirillales; Halomonadaceae |
| MI4-S038 | Ursu Lake (Transylvania) | 1047 | MK504182 | *Halomonas ventosae* | 99.90 | Proteobacteria; Gammaproteobacteria; Oceanospirillales; Halomonadaceae |
| MIII2-S008 | Ursu Lake (Transylvania) | 1008 | MK504183 | *Marinobacter flavimaris/*  *adhaerens* | 100 | Proteobacteria; Gammaproteobacteria; Alteromonadales; Marinobacter_f |
| MI3-H014 | Ursu Lake (Transylvania) | 1123 | MK504184 | *Marinobacter adhaerens* | 99.73 | Proteobacteria; Gammaproteobacteria; Alteromonadales; Marinobacter_f |
| MIII2-S019 | Ursu Lake (Transylvania) | 1039 | MK504185 | *Halomonas arcis* | 99.04 | Proteobacteria; Gammaproteobacteria; Oceanospirillales; Halomonadaceae |
| MII-S016 | Ursu Lake (Transylvania) | 1025 | MK504186 | *Salinivibrio costicola* | 99.90 | Proteobacteria; Gammaproteobacteria; Vibrionales; Vibrionaceae |
| MII3-H007 | Ursu Lake (Transylvania) | 1033 | MK504187 | *Vibrio alginolyticus* | 99.51 | Proteobacteria; Gammaproteobacteria; Vibrionales; Vibrionaceae |
| MII2-S010 | Ursu Lake (Transylvania) | 1049 | MK504188 | *Halomonas alkaliphila* | 99.52 | Proteobacteria; Gammaproteobacteria; Oceanospirillales; Halomonadaceae |
| MVIII2-S004 | Ursu Lake (Transylvania) | 1089 | MK504189 | *Halomonas arcis* | 98.99 | Proteobacteria; Gammaproteobacteria; Oceanospirillales; Halomonadaceae |
| MIII2-S013 | Ursu Lake (Transylvania) | 1057 | MK504190 | *Halomonas arcis* | 98.96 | Proteobacteria; Gammaproteobacteria; Oceanospirillales; Halomonadaceae |
| MIII4-S018 | Ursu Lake (Transylvania) | 1013 | MK504191 | *Bacillus hwajinpoensis* | 99.51 | Firmicutes; Bacilli; Bacillales; Bacillaceae |
| Misz3-S005 | Ursu Lake (Transylvania) | 989 | MK504192 | *Staphylococcus cohnii* | 100 | Firmicutes; Bacilli; Bacillales; Staphylococcaceae |
| MIII4-S017 | Ursu Lake (Transylvania) | 985 | MK504193 | *Bacillus hwajinpoensis* | 99.49 | Firmicutes; Bacilli; Bacillales; Bacillaceae |
| MIII2-S015 | Ursu Lake (Transylvania) | 1073 | MK504194 | *Staphylococcus cohnii* | 100 | Firmicutes; Bacilli; Bacillales; Staphylococcaceae |
| MIII-C004 | Ursu Lake (Transylvania) | 960 | MK504195 | *Salinivibrio costicola* | 99.05 | Proteobacteria; Gammaproteobacteria; Vibrionales; Vibrionaceae |
| MVIII2-H003 | Ursu Lake (Transylvania) | 1056 | MK504196 | *Kocuria palustris* | 100 | Actinobacteria; Actinobacteria_c; Micrococcales; Micrococcaceae |
| MI1-S006 | Ursu Lake (Transylvania) | 1069 | MK504197 | *Pseudoalteromonas agarivorans/*  *distincta* | 99.72 | Proteobacteria; Gammaproteobacteria; Alteromonadales; Pseudoalteromonadaceae |
| MII3-H006 | Ursu Lake (Transylvania) | 1008 | MK504198 | *Pseudoalteromonas aliena* | 99.03 | Proteobacteria; Gammaproteobacteria; Alteromonadales; Pseudoalteromonadaceae |
| MI-C003 | Ursu Lake (Transylvania) | 978 | MK504199 | *Pseudoalteromonas aliena* | 98.88 | Proteobacteria; Gammaproteobacteria; Alteromonadales; Pseudoalteromonadaceae |
| MI1-S002 | Ursu Lake (Transylvania) | 1079 | MK504200 | *Marinobacter excellens* | 99.35 | Proteobacteria; Gammaproteobacteria; Alteromonadales; Marinobacter_f |
| MII3-S032 | Ursu Lake (Transylvania) | 1079 | MK504201 | *Kocuria rosea* | 99.81 | Actinobacteria; Actinobacteria_c; Micrococcales; Micrococcaceae |
| MVIII3-S006 | Ursu Lake (Transylvania) | 1039 | MK504202 | *Halobacillus alkaliphilus* | 99.33 | Firmicutes; Bacilli; Bacillales; Bacillaceae |
| MII-C001 | Ursu Lake (Transylvania) | 1079 | MK504203 | *Pseudoalteromonas aliena* | 99.04 | Proteobacteria; Gammaproteobacteria; Alteromonadales; Pseudoalteromonadaceae |
| MI5-H021A | Ursu Lake (Transylvania) | 1088 | MK504204 | *Halomonas arcis* | 98.99 | Proteobacteria; Gammaproteobacteria; Oceanospirillales; Halomonadaceae |
| MII-C001A | Ursu Lake (Transylvania) | 984 | MK504205 | *Salinivibrio costicola* | 99.17 | Proteobacteria; Gammaproteobacteria; Vibrionales; Vibrionaceae |
| MI5-H021B | Ursu Lake (Transylvania) | 1065 | MK504206 | *Halomonas arcis* | 98.97 | Proteobacteria; Gammaproteobacteria; Oceanospirillales; Halomonadaceae |
| MVIII4-S009 | Ursu Lake (Transylvania) | 1014 | MK504207 | *Halobacillus litoralis* | 99.70 | Firmicutes; Bacilli; Bacillales; Bacillaceae |
| MI1-S001 | Ursu Lake (Transylvania) | 955 | MK504208 | *Vibrio alginolyticus* | 99.37 | Proteobacteria; Gammaproteobacteria; Vibrionales; Vibrionaceae |
| MIII2-H004 | Ursu Lake (Transylvania) | 1048 | MK504209 | *Rhodococcus sovatensis* | 100 | Actinobacteria; Actinobacteria_c; Corynebacteriales; Nocardiaceae |
| MI2-S017 | Ursu Lake (Transylvania) | 1039 | MK504210 | *Halomonas andesensis* | 99.42 | Proteobacteria; Gammaproteobacteria; Oceanospirillales; Halomonadaceae |
| MI1-S004 | Ursu Lake (Transylvania) | 1079 | MK504211 | *Pseudoalteromonas aliena* | 99.04 | Proteobacteria; Gammaproteobacteria; Alteromonadales; Pseudoalteromonadaceae |
| MI3-H013 | Ursu Lake (Transylvania) | 1069 | MK504212 | *Kocuria rosea* | 99.81 | Actinobacteria; Actinobacteria_c; Micrococcales; Micrococcaceae |
| MI-C002B | Ursu Lake (Transylvania) | 1036 | MK504213 | *Vibrio alginolyticus* | 99.42 | Proteobacteria; Gammaproteobacteria; Vibrionales; Vibrionaceae |
| MII-C0010 | Ursu Lake (Transylvania) | 1034 | MK504214 | *Halomonas alkaliphila* | 99.51 | Proteobacteria; Gammaproteobacteria; Oceanospirillales; Halomonadaceae |
| S29 | Slano Kopovo (Vojvodina) | 1036 | MK504215 | *Nesterenkonia halobia* | 97.87 | Actinobacteria; Actinobacteria_c; Micrococcales; Micrococcaceae |
| S28 | Slano Kopovo (Vojvodina) | 1079 | MK504216 | *Hydrogenophaga taeniospiralis* | 98.24 | Proteobacteria; Betaproteobacteria; Burkholderiales; Comamonadaceae |
| S27 | Slano Kopovo (Vojvodina) | 896 | MK504217 | *Micrococcus yunnanensis* | 100 | Actinobacteria; Actinobacteria_c; Micrococcales; Micrococcaceae |
| S26 | Slano Kopovo (Vojvodina) | 1069 | MK504218 | *Azoarcus taiwanensis* | 95.40 | Proteobacteria; Betaproteobacteria; Rhodocyclales; Zoogloeaceae |
| S24 | Slano Kopovo (Vojvodina) | 1027 | MK504219 | *Bacillus zhangzhouensis* | 99.90 | Firmicutes; Bacilli; Bacillales; Bacillaceae |
| S23 | Slano Kopovo (Vojvodina) | 1069 | MK504220 | *Bacillus altitudinis* | 100 | Firmicutes; Bacilli; Bacillales; Bacillaceae |
| S22 | Slano Kopovo (Vojvodina) | 1039 | MK504221 | *Nesterenkonia aurantiaca* | 99.71 | Actinobacteria; Actinobacteria_c; Micrococcales |
| S21 | Slano Kopovo (Vojvodina) | 949 | MK504222 | *Hydrogenophaga taeniospiralis* | 98.73 | Proteobacteria; Betaproteobacteria; Burkholderiales; Comamonadaceae |
| S18 | Slano Kopovo (Vojvodina) | 1099 | MK504223 | *Hydrogenophaga taeniospiralis* | 98.54 | Proteobacteria; Betaproteobacteria; Burkholderiales; Comamonadaceae |
| S15 | Slano Kopovo (Vojvodina) | 999 | MK504224 | *Salipaludibacillus agaradhaerens* | 99.40 | Firmicutes; Bacilli; Bacillales; Bacillaceae |
| S13 | Slano Kopovo (Vojvodina) | 935 | MK504225 | *Bacillus lindianensis* | 99.68 | Firmicutes; Bacilli; Bacillales; Bacillaceae |
| S12 | Slano Kopovo (Vojvodina) | 978 | MK504226 | *Mongoliicoccus roseus* | 99.38 | Bacteroidetes; Cytophagia; Cytophagales; Cyclobacteriaceae |
| S11 | Slano Kopovo (Vojvodina) | 966 | MK504227 | *Bacillus gibsonii* | 99.79 | Firmicutes; Bacilli; Bacillales; Bacillaceae |
| S10 | Slano Kopovo (Vojvodina) | 918 | MK504228 | *Rheinheimera chironomi* | 99.45 | Proteobacteria; Gammaproteobacteria; Alteromonadales; Alishewanella_f |
| S9 | Slano Kopovo (Vojvodina) | 1059 | MK504229 | *Azoarcus taiwanensis* | 94.79 | Proteobacteria; Betaproteobacteria; Rhodocyclales; Zoogloeaceae |
| S8 | Slano Kopovo (Vojvodina) | 1029 | MK504230 | *Hydrogenophaga taeniospiralis* | 98.83 | Proteobacteria; Betaproteobacteria; Burkholderiales; Comamonadaceae |
| S7 | Slano Kopovo (Vojvodina) | 953 | MK504231 | *Vibrio mimicus* | 99.68 | Proteobacteria; Gammaproteobacteria; Vibrionales; Vibrionaceae |
| S5 | Slano Kopovo (Vojvodina) | 970 | MK504232 | *Nesterenkonia aurantiaca* | 99.79 | Actinobacteria; Actinobacteria_c; Micrococcales; Micrococcaceae |
| S2 | Slano Kopovo (Vojvodina) | 771 | MK504233 | *Bacillus zhangzhouensis/*  *safensis* | 100 | Firmicutes; Bacilli; Bacillales; Bacillaceae |
| R39 | Rusanda (Vojvodina) | 1019 | MK504234 | *Roseinatronobacter monicus* | 97.94 | Proteobacteria; Alphaproteobacteria; Rhodobacterales; Rhodobacteraceae |
| R38 | Rusanda (Vojvodina) | 1044 | MK504235 | *Nesterenkonia sandarakina* | 99.71 | Actinobacteria; Actinobacteria_c; Micrococcales; Micrococcaceae |
| R37 | Rusanda (Vojvodina) | 1048 | MK504236 | *Rhodobaca barguzinensis* | 98.19 | Proteobacteria; Alphaproteobacteria; Rhodobacterales; Rhodobacteraceae |
| R36 | Rusanda (Vojvodina) | 959 | MK504237 | *Pararhodobacter aggregans* | 98.81 | Proteobacteria; Alphaproteobacteria; Rhodobacterales; Rhodobacteraceae |
| R35 | Rusanda (Vojvodina) | 1062 | MK504238 | *Porphyrobacter neustonensis* | 99.06 | Proteobacteria; Alphaproteobacteria; Sphingomonadales; Erythrobacteraceae |
| R34 | Rusanda (Vojvodina) | 1017 | MK504239 | *Roseinatronobacter thiooxidans/*  *barguzinensis* | 98.03 | Proteobacteria; Alphaproteobacteria; Rhodobacterales; Rhodobacteraceae |
| R33 | Rusanda (Vojvodina) | 1085 | MK504240 | *Azoarcus taiwanensis* | 94.55 | Proteobacteria; Betaproteobacteria; Rhodocyclales; Zoogloeaceae |
| R32 | Rusanda (Vojvodina) | 1039 | MK504241 | *Nitrincola alkalilacustris* | 100 | Proteobacteria; Gammaproteobacteria; Oceanospirillales; Oceanospirillaceae |
| R30 | Rusanda (Vojvodina) | 957 | MK504242 | *Nesterenkonia aurantiaca* | 99.79 | Actinobacteria; Actinobacteria_c; Micrococcales; Micrococcaceae |
| R29 | Rusanda (Vojvodina) | 914 | MK504243 | *Vibrio metschnikovii* | 99.56 | Proteobacteria; Gammaproteobacteria; Vibrionales; Vibrionaceae |
| R28 | Rusanda (Vojvodina) | 1033 | MK504244 | *Nesterenkonia halobia* | 97.87 | Actinobacteria; Actinobacteria_c; Micrococcales; Micrococcaceae |
| R27 | Rusanda (Vojvodina) | 961 | MK504245 | *Micrococcus yunnanensis* | 100 | Actinobacteria; Actinobacteria_c; Micrococcales; Micrococcaceae |
| R26 | Rusanda (Vojvodina) | 1029 | MK504246 | *Alkalibacterium psychrotolerans* | 99.90 | Firmicutes; Bacilli; Lactobacillales; Carnobacteriaceae |
| R25 | Rusanda (Vojvodina) | 1046 | MK504247 | *Salipaludibacillus neizhouensis* | 98.18 | Firmicutes; Bacilli; Bacillales; Bacillaceae |
| R23 | Rusanda (Vojvodina) | 1019 | MK504248 | *Nesterenkonia halobia* | 97.64 | Actinobacteria; Actinobacteria_c; Micrococcales; Micrococcaceae |
| R22 | Rusanda (Vojvodina) | 1059 | MK504249 | *Vibrio metschnikovii* | 99.62 | Proteobacteria; Gammaproteobacteria; Vibrionales; Vibrionaceae |
| R21 | Rusanda (Vojvodina) | 976 | MK504250 | *Alkalicoccus saliphilus* | 100 | Firmicutes; Bacilli; Bacillales; Bacillaceae |
| R20 | Rusanda (Vojvodina) | 946 | MK504251 | *Nesterenkonia aurantiaca* | 99.79 | Actinobacteria; Actinobacteria_c; Micrococcales; Micrococcaceae |
| R19 | Rusanda (Vojvodina) | 920 | MK504252 | *Bacillus daqingensis* | 99.67 | Firmicutes; Bacilli; Bacillales; Bacillaceae |
| R17 | Rusanda (Vojvodina) | 939 | MK504253 | *Nesterenkonia pannonica* | 99.79 | Actinobacteria; Actinobacteria_c; Micrococcales; Micrococcaceae |
| R14 | Rusanda (Vojvodina) | 803 | MK504254 | *Mongoliibacter ruber* | 98.00 | Bacteroidetes; Cytophagia; Cytophagales; Cyclobacteriaceae |
| R13 | Rusanda (Vojvodina) | 919 | MK504255 | *Alkalimonas amylolytica* | 99.78 | Proteobacteria; Gammaproteobacteria; Alteromonadales; Alishewanella_f |
| R11 | Rusanda (Vojvodina) | 1096 | MK504256 | *Bacillus aurantiacus* | 97.99 | Firmicutes; Bacilli; Bacillales; Bacillaceae |
| R10 | Rusanda (Vojvodina) | 927 | MK504257 | *Bacillus daqingensis/luteus* | 99.68 | Firmicutes; Bacilli; Bacillales; Bacillaceae |
| R5 | Rusanda (Vojvodina) | 957 | MK504258 | *Nesterenkonia sandarakina* | 99.90 | Actinobacteria; Actinobacteria_c; Micrococcales; Micrococcaceae |
| R1 | Rusanda (Vojvodina) | 1072 | MK504259 | *Nesterenkonia aurantiaca* | 99.63 | Actinobacteria; Actinobacteria_c; Micrococcales; Micrococcaceae |
| ZRG14 | Zab-szék (Kiskunság) | 852 | MK504260 | *Bacillus alkalisediminis* | 99.58 | Firmicutes; Bacilli; Bacillales; Bacillaceae |
| ZRG10 | Zab-szék (Kiskunság) | 871 | MK504261 | *Salipaludibacillus halalkaliphilus* | 98.70 | Firmicutes; Bacilli; Bacillales; Bacillaceae |
| ZRG1 | Zab-szék (Kiskunság) | 845 | MK504262 | *Bacillus aurantiacus* | 98.58 | Firmicutes; Bacilli; Bacillales; Bacillaceae |
| ZRA19 | Zab-szék (Kiskunság) | 792 | MK504263 | *Brevundimonas bullata* | 99.75 | Proteobacteria; Alphaproteobacteria; Caulobacterales; Caulobacteraceae |
| ZRA18 | Zab-szék (Kiskunság) | 791 | MK504264 | *Porphyrobacter neustonensis* | 99.24 | Proteobacteria; Alphaproteobacteria; Sphingomonadales; Erythrobacteraceae |
| ZRA14 | Zab-szék (Kiskunság) | 824 | MK504265 | *Micrococcus luteus/ aloeverae/yunnanensis* | 100 | Actinobacteria; Actinobacteria_c; Micrococcales; Micrococcaceae |
| ZRA4 | Zab-szék (Kiskunság) | 852 | MK504266 | *Bacillus alkalisediminis* | 99.88 | Firmicutes; Bacilli; Bacillales; Bacillaceae |
| ZPG13 | Zab-szék (Kiskunság) | 730 | MK504267 | *Salipaludibacillus agaradhaerens* | 100 | Firmicutes; Bacilli; Bacillales; Bacillaceae |
| ZPG11 | Zab-szék (Kiskunság) | 1074 | MK504268 | *Salipaludibacillus halalkaliphilus* | 99.16 | Firmicutes; Bacilli; Bacillales; Bacillaceae |
| ZPG9 | Zab-szék (Kiskunság) | 746 | MK504269 | *Salipaludibacillus halalkaliphilus* | 99.72 | Firmicutes; Bacilli; Bacillales; Bacillaceae |
| ZPG8 | Zab-szék (Kiskunság) | 797 | MK504270 | *Bacillus alkalisediminis* | 100 | Firmicutes; Bacilli; Bacillales; Bacillaceae |
| ZPG7 | Zab-szék (Kiskunság) | 770 | MK504271 | *Bacillus alkalisediminis* | 98.70 | Firmicutes; Bacilli; Bacillales; Bacillaceae |
| ZPG4 | Zab-szék (Kiskunság) | 996 | MK504272 | *Salipaludibacillus agaradhaerens* | 99.90 | Firmicutes; Bacilli; Bacillales; Bacillaceae |
| ZPG1 | Zab-szék (Kiskunság) | 846 | MK504273 | *Salipaludibacillus agaradhaerens* | 99.29 | Firmicutes; Bacilli; Bacillales; Bacillaceae |
| ZPA11 | Zab-szék (Kiskunság) | 733 | MK504274 | *Salipaludibacillus aurantiacus* | 97.95 | Firmicutes; Bacilli; Bacillales; Bacillaceae |
| ZPA9 | Zab-szék (Kiskunság) | 718 | MK504275 | *Bacillus aurantiacus* | 97.63 | Firmicutes; Bacilli; Bacillales; Bacillaceae |
| ZPA7 | Zab-szék (Kiskunság) | 942 | MK504276 | *Anaerobacillus isosaccharinicus* | 99.79 | Firmicutes; Bacilli; Bacillales; Bacillaceae |
| ZPA5 | Zab-szék (Kiskunság) | 844 | MK504277 | *Bacillus kiskunsagensis* | 100 | Firmicutes; Bacilli; Bacillales; Bacillaceae |
| ZPA4 | Zab-szék (Kiskunság) | 842 | MK504278 | *Bacillus aurantiacus* | 98.57 | Firmicutes; Bacilli; Bacillales; Bacillaceae |
| ZPA3 | Zab-szék (Kiskunság) | 844 | MK504279 | *Bacillus lindianensis* | 99.64 | Firmicutes; Bacilli; Bacillales; Bacillaceae |
| ZHG2 | Zab-szék (Kiskunság) | 913 | MK504280 | *Salipaludibacillus agaradhaerens* | 100 | Firmicutes; Bacilli; Bacillales; Bacillaceae |
| ZHA4 | Zab-szék (Kiskunság) | 1125 | MK504281 | *Bacillus lindianensis* | 99.64 | Firmicutes; Bacilli; Bacillales; Bacillaceae |
| ZEG14 | Zab-szék (Kiskunság) | 966 | MK504282 | *Bacillus horikoshii* | 99.17 | Firmicutes; Bacilli; Bacillales; Bacillaceae |
| ZEG12 | Zab-szék (Kiskunság) | 747 | MK504283 | *Salipaludibacillus aurantiacus* | 97.99 | Firmicutes; Bacilli; Bacillales; Bacillaceae |
| ZEG10 | Zab-szék (Kiskunság) | 691 | MK504284 | *Salipaludibacillus aurantiacus* | 97.82 | Firmicutes; Bacilli; Bacillales; Bacillaceae |
| ZEG7 | Zab-szék (Kiskunság) | 880 | MK504285 | *Bacillus populi* | 97.48 | Firmicutes; Bacilli; Bacillales; Bacillaceae |
| ZEG5 | Zab-szék (Kiskunság) | 724 | MK504286 | *Anaerobacillus isosaccharinicus* | 98.61 | Firmicutes; Bacilli; Bacillales; Bacillaceae |
| ZEG4 | Zab-szék (Kiskunság) | 852 | MK504287 | *Bacillus halmapalus* | 97.17 | Firmicutes; Bacilli; Bacillales; Bacillaceae |
| ZEG3 | Zab-szék (Kiskunság) | 1052 | MK504288 | *Bacillus aurantiacus* | 98.57 | Firmicutes; Bacilli; Bacillales; Bacillaceae |
| ZEA13 | Zab-szék (Kiskunság) | 854 | MK504289 | *Bacillus alkalisediminis* | 99.88 | Firmicutes; Bacilli; Bacillales; Bacillaceae |
| ZEA10 | Zab-szék (Kiskunság) | 718 | MK504290 | *Bacillus aurantiacus* | 99.16 | Firmicutes; Bacilli; Bacillales; Bacillaceae |
| ZEA7 | Zab-szék (Kiskunság) | 718 | MK504291 | *Bacillus aurantiacus* | 97.91 | Firmicutes; Bacilli; Bacillales; Bacillaceae |
| ZAG7 | Zab-szék (Kiskunság) | 675 | MK504292 | *Bacillus alkalisediminis* | 100 | Firmicutes; Bacilli; Bacillales; Bacillaceae |
| ZAG5 | Zab-szék (Kiskunság) | 857 | MK504293 | *Bacillus alkalisediminis* | 99.88 | Firmicutes; Bacilli; Bacillales; Bacillaceae |
| ZAG2 | Zab-szék (Kiskunság) | 916 | MK504294 | *Bacillus alkalisediminis* | 99.89 | Firmicutes; Bacilli; Bacillales; Bacillaceae |
| ZAA3 | Zab-szék (Kiskunság) | 849 | MK504295 | *Bacillus lindianensis* | 99.53 | Firmicutes; Bacilli; Bacillales; Bacillaceae |
| ZAA2 | Zab-szék (Kiskunság) | 653 | MK504296 | *Bacillus alkalisediminis* | 100 | Firmicutes; Bacilli; Bacillales; Bacillaceae |
| SRG15 | Sós-ér  (Kiskunság) | 1009 | MK504297 | *Polygonibacillus indicireducens* | 99.40 | Firmicutes; Bacilli; Bacillales; Bacillaceae |
| SRG12 | Sós-ér  (Kiskunság) | 845 | MK504298 | *Bacillus aurantiacus* | 98.58 | Firmicutes; Bacilli; Bacillales; Bacillaceae |
| SRG6 | Sós-ér  (Kiskunság) | 1005 | MK504299 | *Bacillus horikoshii* | 98.50 | Firmicutes; Bacilli; Bacillales; Bacillaceae |
| SRG4 | Sós-ér  (Kiskunság) | 1008 | MK504300 | *Bacillus horikoshii* | 98.61 | Firmicutes; Bacilli; Bacillales; Bacillaceae |
| SRA14 | Sós-ér  (Kiskunság) | 526 | MK504301 | *Bacillus aurantiacus* | 98.85 | Firmicutes; Bacilli; Bacillales; Bacillaceae |
| SRA13 | Sós-ér  (Kiskunság) | 839 | MK504302 | *Bacillus horikoshii* | 99.64 | Firmicutes; Bacilli; Bacillales; Bacillaceae |
| SRA11 | Sós-ér  (Kiskunság) | 852 | MK504303 | *Bacillus aurantiacus* | 97.40 | Firmicutes; Bacilli; Bacillales; Bacillaceae |
| SRA9 | Sós-ér  (Kiskunság) | 845 | MK504304 | *Bacillus aurantiacus* | 97.98 | Firmicutes; Bacilli; Bacillales; Bacillaceae |
| SRA8 | Sós-ér  (Kiskunság) | 825 | MK504305 | *Salipaludibacillus halalkaliphilus* | 99.62 | Firmicutes; Bacilli; Bacillales; Bacillaceae |
| SRA3 | Sós-ér  (Kiskunság) | 801 | MK504306 | *Bacillus horikoshii* | 99.25 | Firmicutes; Bacilli; Bacillales; Bacillaceae |
| SPG13 | Sós-ér  (Kiskunság) | 718 | MK504307 | *Bacillus aurantiacus* | 99.16 | Firmicutes; Bacilli; Bacillales; Bacillaceae |
| SPG11 | Sós-ér  (Kiskunság) | 879 | MK504308 | *Bacillus populi* | 97.36 | Firmicutes; Bacilli; Bacillales; Bacillaceae |
| SPG10 | Sós-ér  (Kiskunság) | 726 | MK504309 | *Bacillus cohnii* | 96.69 | Firmicutes; Bacilli; Bacillales; Bacillaceae |
| SPG9 | Sós-ér  (Kiskunság) | 828 | MK504310 | *Dietzia maris* | 100 | Actinobacteria; Actinobacteria_c; Corynebacteriales; Dietziaceae |
| SPG4 | Sós-ér  (Kiskunság) | 869 | MK504311 | *Salipaludibacillus agaradhaerens* | 100 | Firmicutes; Bacilli; Bacillales; Bacillaceae |
| SPG3 | Sós-ér  (Kiskunság) | 822 | MK504312 | *Mongoliitalea lutea* | 97.48 | Bacteroidetes; Cytophagia; Cytophagales; Cyclobacteriaceae |
| SPA7 | Sós-ér  (Kiskunság) | 848 | MK504313 | *Bacillus aurantiacus* | 98.57 | Firmicutes; Bacilli; Bacillales; Bacillaceae |
| SPA6 | Sós-ér  (Kiskunság) | 962 | MK504314 | *Salipaludibacillus halalkaliphilus* | 99.90 | Firmicutes; Bacilli; Bacillales; Bacillaceae |
| SPA3 | Sós-ér  (Kiskunság) | 741 | MK504315 | *Bacillus lindianensis* | 99.33 | Firmicutes; Bacilli; Bacillales; Bacillaceae |
| SHG1 | Sós-ér  (Kiskunság) | 854 | MK504316 | *Bacillus horikoshii* | 99.41 | Firmicutes; Bacilli; Bacillales; Bacillaceae |
| SHA7 | Sós-ér  (Kiskunság) | 668 | MK504317 | *Bacillus lindianensis* | 99.55 | Firmicutes; Bacilli; Bacillales; Bacillaceae |
| SHA6 | Sós-ér  (Kiskunság) | 859 | MK504318 | *Jeotgalibacillus campisalis* | 99.65 | Firmicutes; Bacilli; Bacillales; Bacillaceae |
| SHA1 | Sós-ér  (Kiskunság) | 1001 | MK504319 | *Bacillus horikoshii* | 98.60 | Firmicutes; Bacilli; Bacillales; Bacillaceae |
| SEG10 | Sós-ér  (Kiskunság) | 718 | MK504320 | *Bacillus aurantiacus* | 97.63 | Firmicutes; Bacilli; Bacillales; Bacillaceae |
| SEG9 | Sós-ér  (Kiskunság) | 747 | MK504321 | *Salipaludibacillus halalkaliphilus* | 98.48 | Firmicutes; Bacilli; Bacillales; Bacillaceae |
| SEG8 | Sós-ér  (Kiskunság) | 852 | MK504322 | *Bacillus aurantiacus* | 98.70 | Firmicutes; Bacilli; Bacillales; Bacillaceae |
| SEG7 | Sós-ér  (Kiskunság) | 900 | MK504323 | *Micrococcus aloeverae* | 100 | Actinobacteria; Actinobacteria_c; Micrococcales; Micrococcaceae |
| SEA12 | Sós-ér  (Kiskunság) | 898 | MK504324 | *Micrococcus luteus* | 100 | Actinobacteria; Actinobacteria_c; Micrococcales; Micrococcaceae |
| SEA10 | Sós-ér  (Kiskunság) | 733 | MK504325 | *Bacillus aurantiacus* | 97.67 | Firmicutes; Bacilli; Bacillales; Bacillaceae |
| SEA9 | Sós-ér  (Kiskunság) | 726 | MK504326 | *Bacillus lindianensis* | 99.59 | Firmicutes; Bacilli; Bacillales; Bacillaceae |
| SEA4 | Sós-ér  (Kiskunság) | 845 | MK504327 | *Bacillus aurantiacus* | 98.58 | Firmicutes; Bacilli; Bacillales; Bacillaceae |
| SEA3 | Sós-ér  (Kiskunság) | 851 | MK504328 | *Jeotgalibacillus campisalis* | 99.65 | Firmicutes; Bacilli; Bacillales; Bacillaceae |
| SEA1 | Sós-ér  (Kiskunság) | 868 | MK504329 | *Bacillus halmapalus* | 98.04 | Firmicutes; Bacilli; Bacillales; Bacillaceae |
| SAA9 | Sós-ér  (Kiskunság) | 674 | MK504330 | *Bacillus lindianensis* | 99.41 | Firmicutes; Bacilli; Bacillales; Bacillaceae |
| SAA7 | Sós-ér  (Kiskunság) | 852 | MK504331 | *Bacillus lindianensis* | 99.41 | Firmicutes; Bacilli; Bacillales; Bacillaceae |
| SAA6 | Sós-ér  (Kiskunság) | 829 | MK504332 | *Bacillus horikoshii* | 100 | Firmicutes; Bacilli; Bacillales; Bacillaceae |
| SAA5 | Sós-ér  (Kiskunság) | 731 | MK504333 | *Salipaludibacillus agaradhaerens* | 100 | Firmicutes; Bacilli; Bacillales; Bacillaceae |

**Supplementary references**

Borsodi A. K., Felföldi T., Máthé I., Bognár V., Knáb M., Krett, G., Jurecska L., Tóth M. E., Márialigeti, K. (2013). Phylogenetic diversity of bacterial and archaeal communities inhabiting the saline Roşu Lake located in Sovata, Romania. *Extremophiles,* **17,** 87-98.

Felföldi T., Ramganesh S., Somogyi B., Krett G., Jurecska L., Szabó A., Vörös L., Márialigeti K., Máthé I. (2016). Winter planktonic microbial communities in highland aquatic habitats. *Geomicrobiology Journal,* **33,** 494-504.

Máthé I., Borsodi A. K., Tóth E. M., Felföldi T., Jurecska L., Krett, G., Kelemen Zs., Elekes E., Barkács K., Márialigeti, K. (2014). Vertical physico-chemical gradients with distinct microbial communities in the hypersaline and heliothermal Lake Ursu Lake (Sovata, Romania). *Extremophiles,* **18,** 501-514.
